# A New and Safe Method of Delivery in Placenta Prævia

**Published:** 1899-08-26

**Authors:** John Mooney

**Affiliations:** Manchester


### Hospital Clinics and Medical Progress.

### A NEW AND SAFE METHOD OF DELIVERY IN PLACENTA PRÆVIA.

By John Mooney, M.B., Ch.B., Manchester.

After placenta prævia has been diagnosed, and it has been decided to induce labour, the process consists in: (1) The arrangement of the child by external abdominal manipulation, so that its long axis shall be in the same direction as the long axis of the uterus of the mother, if it is not already so directed; (2) the application of a firm binder, capable of being tightened, and so placed as to press one pole of the child down against the placenta at the os uteri; (3) the perforation of the placenta; (4) the perforation of the membranes at the same spot as the perforation in the placenta; (5) the slight enlargement of these apertures; (6) the delivery of the child through them; and, finally (7), the expression of the placenta itself. The process is applicable to any case of placenta prævia, whether central or marginal, and can be carried out whether pains have commenced or not, and whether there is hæmorrhage or not. If convenient it is, perhaps, best to induce labour about the time of some gush of hæmorrhage, as the os is then generally more dilated than at other times.

The presentation of the child can generally be easily made out abdominally, since the placenta being placed low down permits the fœtus to be more easily felt through the fundus uteri. Some idea of the presentation can also be obtained from the position of the heart sounds. If the presentation is transverse make it longitudinal by pressing the opposite poles of the child in different directions, the operator's hands being both on the abdomen. Before anything is done a stout binder, about ten inches wide and five or six feet long, should be adjusted round the patient's abdomen so that its upper margin dips in between the lower ribs and the fundus uteri. The ends of the binder should be long enough to cross one another behind the patient's back at a lower level than the part of the binder which is in front of the abdomen, so that tightening the binder brings downward pressure to bear on the fundus. The ends of the binder may be crossed or screwed, and should be held with a firm grip. They should not be pinned or tied, because this prevents the speedy application of extra force on the binder when necessary. It is almost possible to bring the os uteri to the vulva by pressure exerted in this way.

Having adjusted the binder, and having seen that the presentation is all right, and that there is no abnormal obstruction to delivery except the placenta, give one dram of liquid extract of ergot. Then pass the forefinger of the right hand into the os until it touches the soft spongy placenta. Through this the finger is gradually worked and pushed until the tough membranes covering the internal surface of the placenta are reached. (If the fœtal head is beyond these it is very easy to mistake them for the surface of the head itself.) The perforation of the placenta in this way causes very little bleeding, probably because the placental vessels are more pushed aside than torn. The opening in the placenta is then enlarged with the finger and the membranes at once ruptured. Mere puncture of these membranes does not make them rupture, seeing that they are bound down to the placenta and have branches of the umbilical arteries of the cord ramifying in them. The aperture in placenta and membranes should be about the size of a florin. At this stage it is necessary to tighten the binder. There is no hæmorrhage because the binder keeps the fœtal head well pressed down against the perforated placenta, compressing it against the uterine wall. The caput succedaneum sticking into the aperture also prevents bleeding. The os, and with it the placenta, now gradually dilate under the influence of the pains and the pressure of the binder. The child's head comes through the opening in the placenta, descends along the vaginal canal, and is born. There are generally a few clots about the child's neck. There is a little hæmorrhage after the child is delivered until the placenta has come away, which should be expressed immediately after delivery. When the placenta has come away the bag of membranes is seen to be unruptured, the only opening being in the placenta. This artificial aperture is not above two inches in diameter; on inspection afterwards it seems inconceivable that a child could have been born through it.

Liquid extract of ergot should be given in dram doses every hour until pains come on, or until five drams have been given. The first dose should, if possible, be given half-an-hour before perforating the placenta, previously making sure that there is no abnormal obstruction; or ergotin may be injected instead.

The whole labour may last several hours. The advantages of the method appear to be: (1) That the risk to the mother is very much decreased because there is so little hæmorrhage, since the uterine vessels are never free from pressure; (2) it is not necessary to cause her pain and danger of sepsis by introducing the hand into the vagina and part of it into the uterus, as it is in the method of detaching the placenta and then turning; (3) there is no risk of rupture of uterus or shock, as in the old method of turning; (4) it acts very satisfactorily in centrally-situated placenta prævia, formerly considered the most dangerous kind.

The above method has been tried in three cases, and was successful in all.

